# Assessment of stroke volume variation for prediction of fluid responsiveness using the modified FloTrac™ and PiCCOplus™ system

**DOI:** 10.1186/cc6933

**Published:** 2008-06-20

**Authors:** Christoph K Hofer, Alban Senn, Luc Weibel, Andreas Zollinger

**Affiliations:** 1 Institute of Anaesthesiology and Intensive Care Medicine, Triemli City Hospital, Birmensdorferstrasse, CH-8063 Zurich, Switzerland; 2 Department of Internal Medicine, Triemli City Hospital, Birmensdorferstrasse, CH-8063 Zurich, Switzerland

## Abstract

**Introduction:**

Stroke volume variation (SVV) has repeatedly been shown to be a reliable predictor of fluid responsiveness. Various devices allow automated clinical assessment of SVV. The aim of the present study was to compare prediction of fluid responsiveness using SVV, as determined by the FloTrac™/Vigileo™ system and the PiCCOplus™ system.

**Methods:**

In patients who had undergone elective cardiac surgery, SVV_FloTrac _was determined via radial FloTrac sensor, and SVV_PiCCO _and pulse pressure variation were assessed via a femoral PiCCO catheter. Stroke volume was assessed by transpulmonary thermodilution. All variables were recorded before and after a volume shift induced by a change in body positioning (from 30° head-up position to 30° head-down position). Pearson correlation, *t*-test, and Bland-Altman analysis were performed. Area under the curve was determined by plotting receiver operating characteristic curves for changes in stroke volume in excess of 25%. *P *< 0.05 was considered statistically significant.

**Results:**

Body positioning resulted in a significant increase in stroke volume; SVV_FloTrac _and SVV_PiCCO _decreased significantly. Correlations of SVV_FloTrac _and SVV_PiCCO _with change in stroke volume were similar. There was no significant difference between the areas under the curve for SVV_FloTrac _and SVV_PiCCO_; the optimal threshold values given by the receiver operating characteristic curves were 9.6% for SVV_FloTrac _(sensitivity 91% and specificity 83%) and 12.1% for SVV_PiCCO _(sensitivity 87% and specificity 76%). There was a clinically acceptable agreement and strong correlation between SVV_FloTrac _and SVV_PiCCO_.

**Conclusion:**

SVVs assessed using the FloTrac™/Vigileo™ and the PiCCOplus™ systems exhibited similar performances in terms of predicting fluid responsiveness. In comparison with SVV_PiCCO_, SVV_FloTrac _has a lower threshold value.

## Introduction

Fluid administration in critically ill patients is typically performed to increase cardiac preload, followed by a raise in cardiac output. However, studies conducted during the past few years have shown that about 50% of critically ill patients do not exhibit the desired effect (they are not fluid responsive) [1]. Thus, we require an accurate and reliable technique to guide fluid management. Pressure preload variables (central venous pressure and pulmonary capillary wedge pressure), which continue to be used, often fail to provide reliable information regarding cardiac preload [2] and are incapable of predicting cardiac response to fluid therapy [3]. On the other hand, the volumetric preload variables that are assessed by transpulmonary thermodilution may better reflect left ventricular preload [4], but they do not allow assessment of fluid responsiveness [3,5].

As an alternative to these static variables, a dynamic approach may be used in the form of preload monitoring to guide fluid therapy. With passive leg rising in spontaneously breathing patients, the heart's reaction (increased venous return) can be assessed without any fluid administration [6], and in mechanically ventilated patients the interaction between heart and lung can be used to predict fluid responsiveness [7].

Different, less invasive haemodynamic monitoring systems based on arterial pulse contour analysis allow stroke volume variation (SVV) to be tracked continuously. SVV assessed using the PiCCO_plus_™ system (Pulsion Medical Systems, Munich, Germany; SVV_PiCCO_) has repeatedly been shown to predict fluid responsiveness well in various clinical settings[3,8-11], whereas only sparse data are available for SVV determined using the recently introduced FloTrac™/Vigileo™ system (Edwards Lifesciences, Irvine, CA, USA; SVV_FloTrac_). In a study conducted by de Waal and coworkers [12], SVV_FloTrac _failed to predict fluid responsiveness. This finding may be attributable to the initial version of the device's software, adapting vascular compliance every 10 minutes. Limited accuracy in assessment of cardiac output (and thus of stroke volume) could be achieved using this early software version [13]. Modification to the software (reduction in the time window for vascular adjustment to 1 minute) resulted in improved accuracy in measuring cardiac output [14].

The aim of the present study was to compare SVV_FloTrac _with SVV_PiCCO _during a volume shift manoeuvre (by changing body positioning) in patients who had undergone elective off-pump coronary artery bypass grafting.

## Materials and methods

### Patients and setting

Patients undergoing elective coronary artery bypass grafting gave written, informed consent to participate in the study, which was approved by the local ethics committee. Exclusion criteria were reduced left and right ventricular function (ejection fraction < 40%), preoperative dysrhythmia, intracardiac shunt, pulmonary arterial hypertension, severe arterial occlusion disease and body weight under 40 kg. We calculated that a sample size of 40 patients was necessary, based on an expected standard deviation of 5% in SVV values and an expected difference between SVV assessed using the different systems in the same range (α = 0.05 and power = 0.9).

### Routine perioperative management

Perioperative management was in accordance with institutional standards. Routine monitoring (Philips IntelliVue™ Monitoring; Philips Medical Systems, Andover, MA, USA) during the perioperative period included pulse oxymetry, five-lead electrocardiography, and invasive blood pressure measurement (via a peripheral radial arterial) and central venous pressure (assessed using standard transducers; Truewave™ PX, Edwards Lifesciences). During the time when the study was performed, continuous cardiac output monitoring with the PiCCOplus™ system was routine in the selected patient group. A 4F thermistor-tipped arterial catheter (Pulsiocath™ thermodilution catheter) was inserted into the left femoral artery, and its tip was advanced to the abdominal aorta and connected to a stand-alone PiCCOplus™ monitor (software version 6.0; Pulsion Medical Systems, Munich, Germany). Continuous cardiac output measurement was initiated after initial calibration of the system by injection three times of 20 ml ice-cold normal saline into the central venous catheter (transpulmonary thermodilution).

### Study protocol

Measurements were started during the postoperative period after transfer of patients to the intensive care unit. The patients remained sedated during the study period using propofol (1 to 2 mg/kg per hour) and remifentanil (2 to 5 μg/kg per hour); rocuronium (0.2 to 0.5 mg/kg per hour) was given for neuromuscular blockade. The patients were mechanically ventilated using a volume-controlled mode (tidal volume 8 to 10 ml/kg, respiratory frequency 12 breaths/minute, positive end-expiratory pressure 5 cmH_2_O, peak inspiratory pressure 23 ± 3 cmH_2_O) in order to achieve normoventilation (partial carbon dioxide tension 4 to 4.5 kPa). Weaning from the ventilator was begun after completion of the study protocol. Mean arterial pressure was maintained between 65 and 75 mmHg by adjusting the patient's noradrenaline (norepinephrine) dose (0 to 10 μg/minute). A regular heart rhythm was maintained in all patients by fixed external pacing at a heart rate between 80 to 90 beats/minute.

The femoral PiCCO™ catheter was connected to a standalone PiCCOplus™ monitor (computer version 6.0.1; Pulsion Medical Systems) and recalibrated in accordance with the manufacturer's instruction. A FloTrac™ sensor kit was connected to the radial arterial line and coupled to the Vigileo™ monitor (software version 1.07; Edwards Lifesciences). Individual patient data (age [years], sex, body weight [kg] and height [cm]) were entered. After checking the fidelity of the arterial line waveform, the system was zeroed at mid-axillary level to ambient pressure and measurements were initiated. Measurements were performed 15 minutes before and after a volume shift induced by a change to body positioning (from 30° head-up position to 30° head-down position). At both measurement time points the following continuous haemodynamic variables were recorded: mean arterial pressure, heart rate and central venous pressure (CVP). Also recorded were stroke volume (SV) and SVV_FloTrac _determined using the FloTrac™/Vigileo™ system, and SV, SVV_PiCCO _and pulse pressure variation (PPV) determined using the PiCCOplus™ system. Immediately after, triplicate transpulmonary thermodilution measurements of 20 ml normal iced saline solution were performed in order to determine cardiac output, SV and global end-diastolic volume (GEDV).

### SVV determination

SVV is calculated from percentage changes in SV during the ventilatory cycle. SV is assessed by the FloTrac™/Vigileo™ and the PiCCOplus™ system using different proprietary algorithms, which have been described in detail elsewhere [14-16]. Briefly, calculation of SV by the FloTrac™/Vigileo™ system is based on the contribution of pulse pressure to SV being proportional to the standard deviation of arterial pulse pressure. In order to determine SV, the influences of vascular resistance and compliance on SV are considered using manually entered patient data and pulse wave analysis. In contrast, the PiCCOplus™ method relies on work conducted by Wesseling and coworkers [17], calculating cardiac output by measuring the area under the systolic part of the arterial pressure wave form and dividing this area by the aortic impedance. For adequate determination of SV and adjustment of individual aortic compliance, however, calibration by transpulmonary thermodilution is required.

SVV is assessed by both systems using the following equation: SVV (%) = (SV_max _- SV_min_)/SV_mean_. SV_max_, SV_min _and SV_mean _are determined by the FloTrac™/Vigileo™ system during a time window of 20 seconds. The system can detect and eliminate premature ventricular contractions or other arrhythmias for assessment of SVV. The PiCCOplus™ system measures SV_max _and SV_min _as mean values of the four extreme values of SV during a measurement period of 30 seconds and SV_mean _is recorded as the average value during this time period. In addition, using the PiCCO_plus _system, PPV can be determined during the same time interval [3].

### Data analysis

All haemodynamic variables were recorded as mean values of three repeated measurements. Statistical analysis was performed using Statview^® ^5.01 Software (SAS Institute Inc. Cary, NC, USA) and SPSS^® ^10.0 (SPSS^® ^Inc., Chicago, IL, USA). Student's *t*-test was used for comparison of haemodynamic data before and after change in body position. Pearson's correlation between stroke volume changes and changes in the various haemodynamic variables was established. Prediction of fluid responsiveness based on SVV_FloTrac_, SVV_PiCCO_, PPV and static preload variables (CVP and GEDV) was tested by calculating the AUC (area under the receiver operating characteristic [ROC] curve) for a SV increase greater than 25%. Threshold values for SVV_FloTrac_, SVV_PiCCO _and PPV were determined by considering values that yielded the greatest sensitivity and specificity. Based on these threshold values, the positive and negative predictive values for all dynamic variables were calculated. Additionally, regression analysis was performed for preload variables and SV changes. Comparison of SVV_FloTrac _with SVV_PiCCO _was done by Bland-Altman analysis and Pearson's correlation. ROC curves were compared in accordance with the method established by Hanley and coworkers [18]. Linear correlations were compared using Fisher z-transformation and Hotelling-Williams test. Identification of patients with a SV increase greater than 25% using SVV_FloTrac _and SVV_PiCCO _was compared by χ^2 ^test. *P *< 0.05 was considered to be statistically significant. Unless otherwise stated, data are presented as mean ± standard deviation.

## Results

40 patients (american society of anesthesiologists risk classification III, female/male ratio 1/4, age 66.5 ± 9.2 years, left ventricular ejection fraction 56.1 ± 10.0%) were enrolled in this study. During the study period, no significant changes in ventilatory tidal volumes or peak inspiratory pressure were identified.

**Figure 1:**
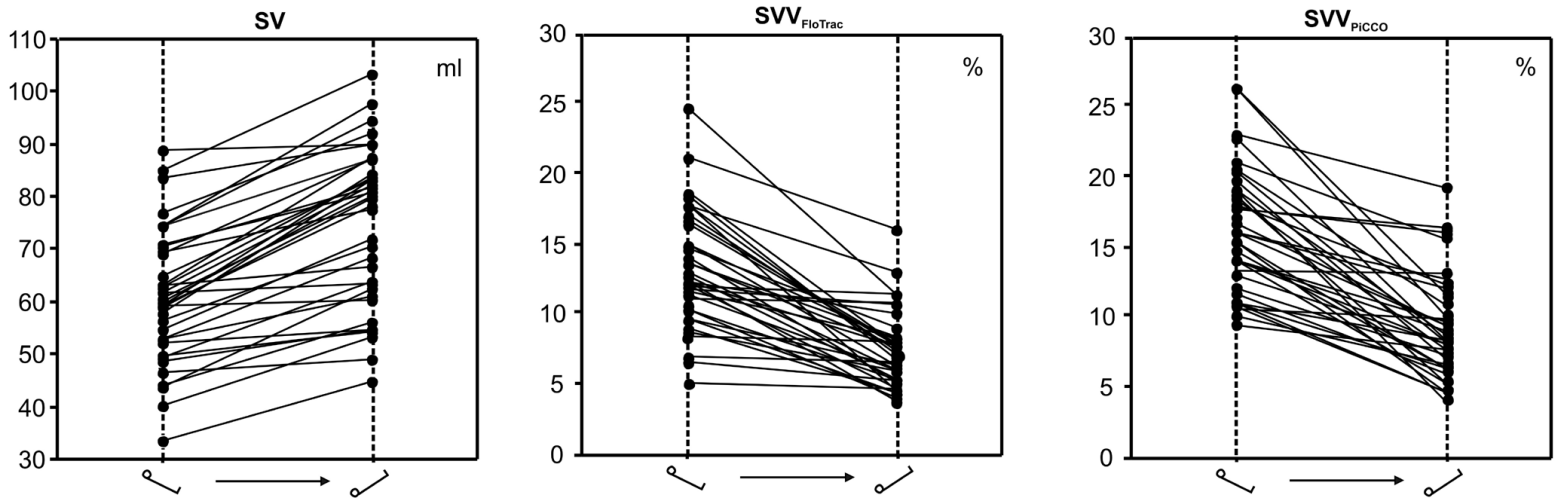
Individual responses of SV, SVV_FloTrac _and SVV_PiCCO _to 30° head-down positioning. SV, stroke volume; SVV, stroke volume variation.

Change in body position from 30° head-up to 30° head-down resulted in significantly increased SV, GEDV and CVP, whereas SVV_FloTrac_, SVV_PiCCO _and PPV significantly decreased (Table 1 and Figure 1). The mean increase in SV was 24 ± 14%. Twenty-three patients (58%) had an increase in SV of greater than 25% (mean change 34 ± 9%). A SV increase of under 10% was observed in eight patients (20%; mean change = 5 ± 3%).

**Table 1:** Haemodynamic data

Parameter	30° head-up	30° head-down	*P*
HR (beats/minute)	87 ± 9	87 ± 8	0.297
MAP (mmHg)	70 ± 9	80 ± 10	<0.001
SVR (dyne·second/cm^5^)	955 ± 178	898 ± 199	0.032
CO (l/minute)	5.0 ± 0.8	6.3 ± 1.2	<0.001
SV (ml)	60 ± 12	76 ± 14	<0.001
SVV_FloTrac _(%)	14 ± 4	8 ± 3	<0.001
SVV_PiCCO _(%)	16 ± 5	9 ± 4	<0.001
PPV (%)	15 ± 6	8 ± 4	<0.001
CVP (mmHg)	7 ± 3	11 ± 3	<0.001
GEDV (ml)	1214 ± 354	1356 ± 392	<0.001

CO, cardiac output; CVP, central venous pressure; GEDV, global end-diastolic volume; HR, heart rate; MAP, mean arterial pressure; PPV, pulse pressure variation; SV, stroke volume; SVR, systemic vascular resistance; SVV, stroke volume variation.

**Figure 2:**
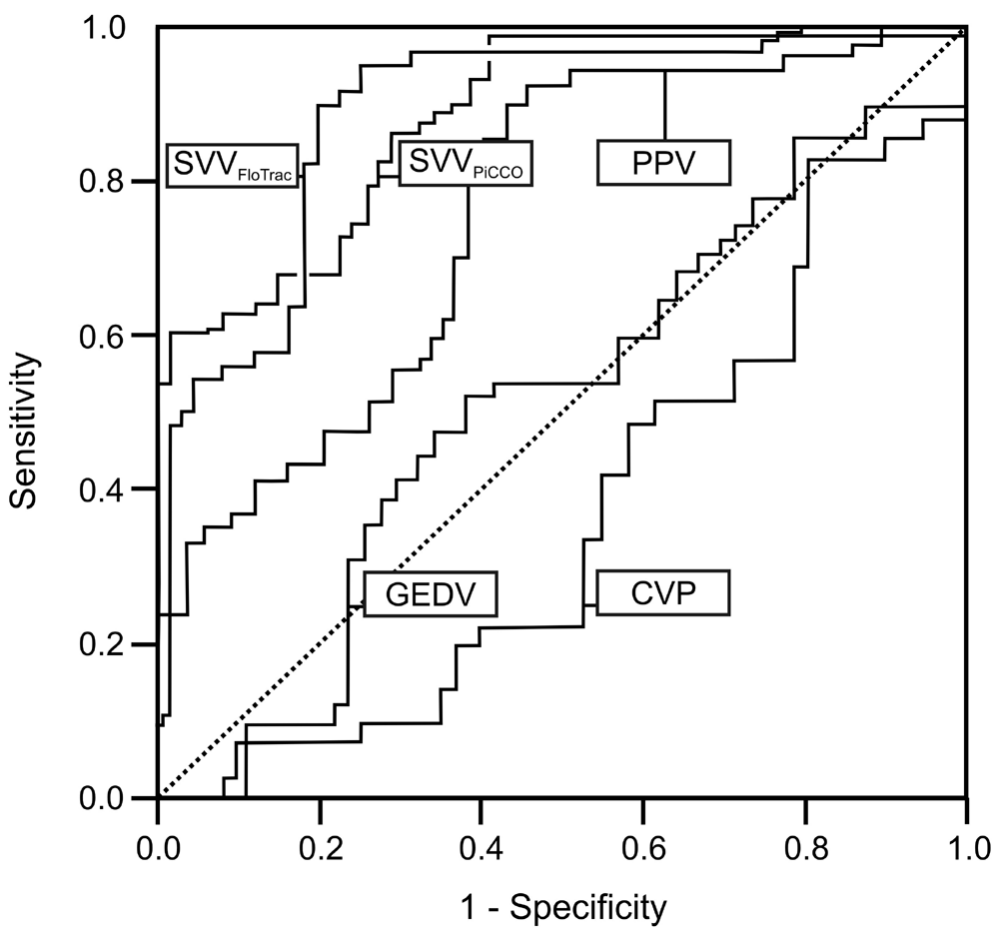
Prediction of fluid responsiveness to SV changes > 25% induced by 30° head-down positioning. CVP, central venous pressure; GEDV, global end-diastolic volume; PPV, pulse pressure variation; SVV, stroke volume variation.

**Figure 3:**
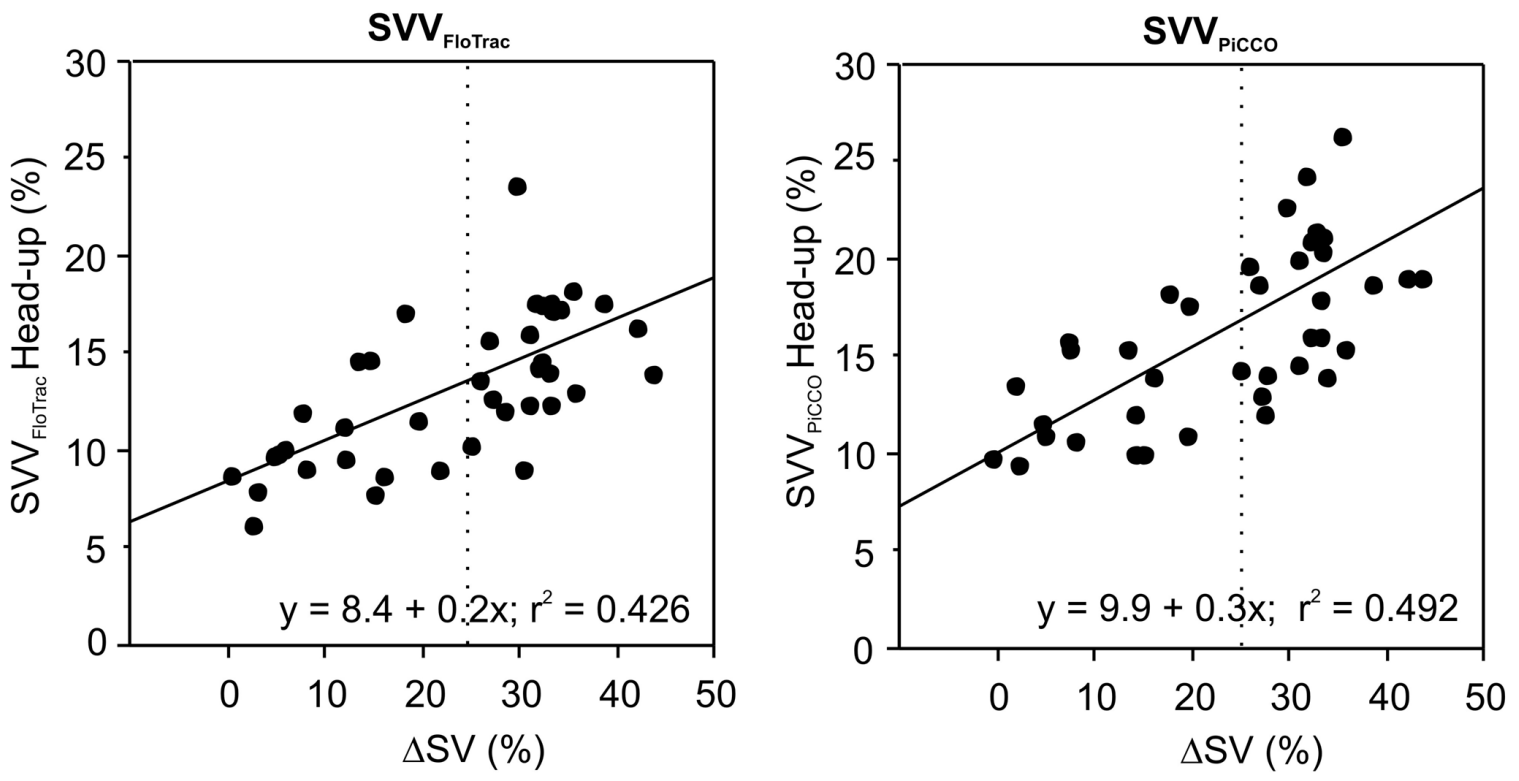
Prediction of fluid responsiveness: Pearson correlations. Shown are Pearson correlations between stroke volume variation (SVV) assessed using the FloTrac™/Vigileo™ and the PiCCOplus™ systems in head-up position and stroke volume (SV) changes induced by 30° head-down positioning. ΔSV, stroke volume change (%).

For patients with an increase in SV of greater than 25%, baseline SVV_FloTrac _and SVV_PiCCO _were 16 ± 4% and 19 ± 5%, respectively. In patients with an increase in SV of under 10%, baseline SVV_FloTrac _and SVV_PiCCO _were 9 ± 2% and 11 ± 3%, respectively. Results of ROC curve and linear regression analyses for the prediction of SV changes induced by altered body positioning are summarized in Figure 2 and Table 2. There was no significant difference between AUCs with respect to identifying a SV increase of more than 25% for SVV_FloTrac _and SVV_PiCCO _(Table 3). Optimal threshold values given by the ROC curves were 9.6% for SVV_FloTrac _(sensitivity 91% and specificity 83%) and 12.1% for SVV_PiCCO _(sensitivity 87% and specificity 76%). Based on these threshold values, positive and negative predictive values were 80% and 92% for SVV_FloTrac_, respectively; corresponding values for SVV_PiCCO _were 77% and 90%. There was no significant difference between identification of patients with a SV increase of more than 25% using SVV_FloTrac _and SVV_PiCCO _(*P *= 0.523). Correlations between SVV_FloTrac _and SVV_PiCCO _and changes of SV (ΔSV) were similar (Tables 2 and 4, and Figure 3).

**Table 2:** Prediction of stroke volume responsiveness

Baseline haemodynamic indices	ROC curves: predicting ΔSV > 25%			Pearson's correlation: baseline haemodynamic indices versus ΔSV	
	
	AUC	95% CI	*P*^a^	*r*^2^	*P*
SVV_FloTrac_	0.824	0.680 to 0.967	<0.001	0.426	<0.001
SVV_PiCCO_	0.858	0.745 to 0.971	<0.001	0.492	<0.001
PPV	0.718	0.578 to 0.898	0.011	0.334	<0.001
GEDV	0.509	0.323 to 0.695	0.632	0.061	0.580
CVP	0.299	0.134 to 0.465	0.924	0.010	0.730

^a^Comparison with AUC = 0.5. AUC, area under the ROC curve; CI, confidence interval; CVP, central venous pressure; GEDV, global end-diastolic volume; PPV, pulse pressure variation; *r*^2^, Pearson correlation coefficient; ROC, receiver operating characteristic; ΔSV, % change in stroke volume; SVV, stroke volume variation.

**Table 3:** *P *values for comparisons of ROC curves

	SVV_FloTrac_	SVV_PiCCO_	PPV	GEDV
SVV_PiCCO_	0.616			
PPV	0.039	0.042		
GEDV	0.015	0.009	0.119	
CVP	<0.001	<0.001	0.042	0.233

CVP, central venous pressure; GEDV, global end-diastolic volume; PPV, pulse pressure variation; ROC, receiver operating characteristic; SVV, stroke volume variation.

**Table 4:** Pearson correlation coefficients (*r*^2^)

	SVV_FloTrac_	SVV_PiCCO_	PPV	GEDV
SVV_PiCCO_	0.749			
PPV	0.295	0.171		
GEDV	0.016	0.008	0.049	
CVP	0.002	0.001	0.026	0.368

CVP, central venous pressure; GEDV, global end-diastolic volume; PPV, pulse pressure variation; ROC, receiver operating characteristic; SVV, stroke volume variation.

The AUCs for PPV, GEDV and CVP were significantly lower than the AUCs for SVV_FloTrac _and SVV_PiCCO _(Table 3). The optimal threshold value was 15.4% for PPV, yielding a sensitivity of 60% and a specificity of 62%, and negative and positive predictive values being 68% and 60%, respectively. Moreover, the correlation between PPV with ΔSV was significant, whereas no significant correlations of GEDV and CVP with ΔSV could be established (Tables 2 and 4).

**Figure 4:**
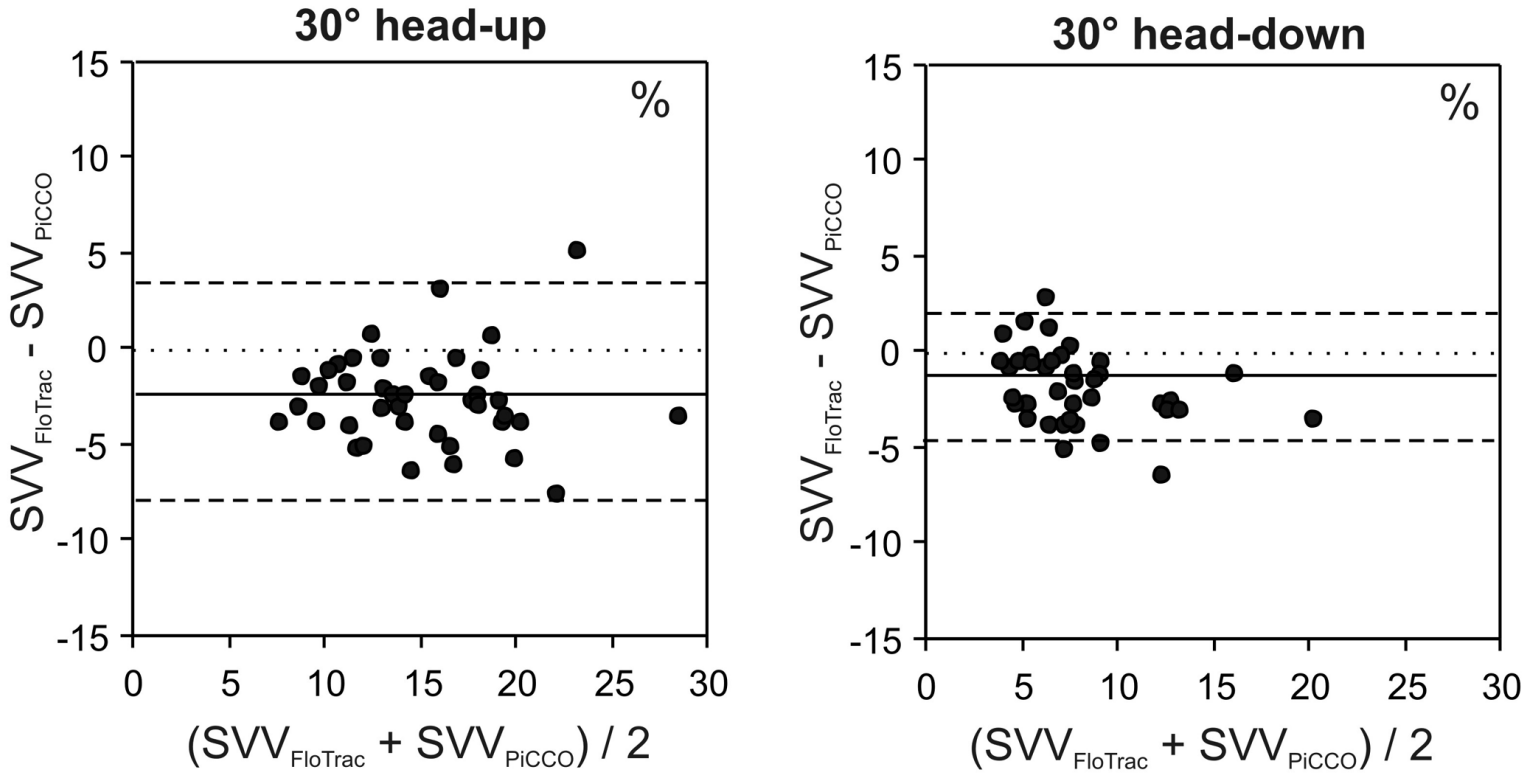
Bland-Altman analysis. Presented is a Bland-Altman analysis comparing stroke volume variation (SVV) assessed using the FloTrac™/Vigileo™ and the PiCCOplus™ system in 30° head-up and 30° head-down positions. 30° head-up: mean bias ± 2 standard deviations (SD; limits of agreement) = -2.5 ± 6.1%; 30° head-down: mean bias ± 2SD = -1.5 ± 3.6%.

Bland-Altman analysis (SVV_FloTrac _- SVV_PiCCO_) revealed a mean bias ± 2 standard deviations (limits of agreement) of -2.5 ± 6.1% for head-up measurements and -1.5 ± 3.6% for head-down measurements (Figure 4). Correlation coefficients for SVV_FloTrac _versus SVV_PiCCO _(*r*^2^) were 0.612 and 0.757 for head-up and head-down measurements, respectively.

## Discussion

SVVs as assessed using the modified FloTrac™/Vigileo™ and the PiCCOplus™ systems were comparable predictors of SV changes related to a fluid shift manoeuvre in patients who had undergone off-pump coronary artery bypass grafting. There was clinically acceptable agreement and a strong correlation between SVV assessed using the two devices. However, in comparison with SVV_PiCCO_, it must be noted that the SVV_FloTrac _device has a lower threshold value.

Differences between the devices in terms of absolute values, and thus their threshold values, may primarily be explained by different signal detection sites and specific differences in the measurement techniques, despite the fact that both systems use a similar SVV algorithm. Both the FloTrac/Vigileo™ and the PiCCOplus™ system are based on analysis of pulse pressure which is the result of the interaction between SV and the systemic vascular system. Therefore, vascular resistance and compliance at the site of signal detection must be considered in order to determine SV and SVV reliably. The FloTrac/Vigileo™ system assesses SV typically using signal detection via a peripheral radial artery. It analyzes the impact of vascular tone on pressure during a period of 20 seconds, and adjusts for actual vascular tone at intervals of 1 minute based on wave form analysis and patient characteristics. In contrast, with the PiCCOplus™ system aortic pressure wave forms are recorded usually via a specific thermistor-tipped arterial catheter in the femoral artery. SV is then calculated using an algorithm that assesses the area under the systolic part of the pressure wave form beat-to-beat after calibration by transpulmonary thermodilution [16].

Only limited data are available regarding SVV as assessed using the FloTrac/Vigileo™ system. Recently, de Waal and coworkers [12] demonstrated that SVV_FloTrac _was unable to predict fluid responsiveness in cardiac surgery patients; their findings contradict those of the present study. However, de Waal and coworkers used the FloTrac/Vigileo™ system with an early version of the software, which employed a time window for adjustment to vascular tone of 10 minutes. Based on the limited accuracy of cardiac output assessment observed with this early software version [13], the algorithm has been modified and – a major change – the time window has been reduced to 1 minute. These modifications resulted in improved measurement of cardiac output [14], and in the present study, in which we used a new software version, we demonstrated that SVV_FloTrac _is useful for predicting fluid responsiveness.

The SVV_PiCCO _findings in the present study are in agreement with those of a variety of previous investigations. SVV_PiCCO _could predict fluid responsiveness in patients with severe sepsis [8], in those undergoing neurosurgery [19] and in cardiac surgery patients with preserved [3,9,10] as well as reduced left ventricular function [9]. Interestingly, Wiesenack and coworkers [20] identified no correlation between SVV_PiCCO _and SV changes during a fluid trial using an older version of the PiCCO algorithm. However, favourable results however were later reported by the same study group [11] using a PiCCO algorithm that has been modified to address better aortic compliance in the individual patient.

Despite the clinical importance of SVV threshold values, little information is available in the literature. A SVV threshold value represents the 'trigger' value at which initiation of fluid replacement therapy is expected to result in a positive cardiac response. ROC analyses performed thus far have revealed that SVV_PiCCO _at the threshold value of 9.5% results in a stepwise SV index increase of 5% or more in studies involving incremental volume administration [9,19]. In a previous study [3] we observed that a SVV_PiCCO _threshold value of 12.5% resulted in an increase in SV index of 25% or more after fluid loading. In the present study, by inducing a fluid shift by altering body positioning, a comparable SVV_PiCCO _threshold value (12.1%) for SV increase greater than 25% was identified, whereas the SVV threshold value for the FloTrac/Vigileo™ system was 9.6%.

Variations in SV are followed by concomitant changes in arterial pressure, which can be assessed by measuring PPV. In addition to SVV, the PiCCO_plus _system allows automated assessment of PPV. Reuter and coworkers [21] found a close correlation between PPV and SVV. Moreover, in a previous study [3], we demonstrated that both SVV and PPV were similarly able to predict fluid responsiveness. However in the present study the predictive performance of PPV was inferior to those of SVV_Flotrac _and SVV_PiCCO_; it is likely that this finding is mainly accounted for by the study design. In this study preload changes were induced by changes in body positioning, whereas in the previous study [3] a fluid trial using hetastarch solution was performed. Body positioning might have influenced vascular tone, and PPV is known to be more susceptible to changes in vasomotor tone than SVV [22].

In contrast to SVV and PPV, conventional static preload parameters assessed in this study failed to predict fluid responsiveness, confirming the findings of previous work related to this issue [3,23]. CVP, the standard pressure preload variable, did not adequately reflect preload status [2,4] and is therefore unsuitable for predicting ventricular response to fluid loading. GEDV, a volumetric preload variable, more adequately reflected changes in cardiac preload [4,24], but this parameter also failed to predict the reaction of the heart to fluid loading.

When interpreting of the data presented in this study, some methodological aspects and limitations must be considered. First, we induced a fluid shift by changing body positioning and not by subjecting patients to a 'real' fluid challenge. Changes in body positioning might have resulted in alterations to systemic vascular resistance based on sympathetic activity and not on changes in preload in isolation. Thus, a fluid shift due to changed body positioning and a true episode of hypovolaemia followed by intravenous fluid administration are not necessarily comparable. However, the sympathetic effect was minimized by using adequate sedation and analgesia in all patients during the study. Furthermore, the significant increase in GEDV observed after changed body positioning indicates an increase in preload. Moreover, body positioning is a typical manoeuvre for assessing fluid responsiveness [1,6]. In addition, a similar approach has previously been used to determine fluid responsiveness of SVV_PiCCO _[10], and we previously observed a similar haemodynamic pattern and comparable threshold value for SVV_PiCCO _in a true fluid challenge [3]. Second, mechanical ventilation and a regular heart rhythm are prerequisites for reliable SVV assessment. In order to satisfy these requirements, patients were fully sedated, neuromuscular blockade was used and fixed cardiac pacing was applied in all patients. However, pacing resulted in a lack of heart rate response to the fluid shift. Moreover, it may not be possible to achieve these optimal conditions in clinical practice.

Thus, our findings may not be directly applicable to other situations or to patients other than the population studied (patients with preserved left ventricular function after elective cardiac surgery). Therefore, further evaluation of SVV_FloTrac _using intravenous fluid administration under different conditions and populations of critically ill patients is needed.

## Conclusion

In conclusion, SVV assessed using the FloTrac™/Vigileo™ and the PiCCOplus™ system exhibited comparable performance in terms of predicting fluid responsiveness. However, in comparison with SVV_PiCCO_, SVV_FloTrac _has a lower threshold value.

## Key messages

• Fluid responsiveness can reliably be assessed using SVV determined using the modified FloTrac™/Vigileo™ system.

• SVV assessed using the modified FloTrac™/Vigileo™ and the PiCCOplus™ systems exhibited similar performance.

• The threshold value was lower for SVV_FloTrac _(9.6%) than for SVV_PiCCO _(12.1%).

• Compared with SVV, PPV was inferior in terms of predicting fluid responsiveness; static preload variables assessed in the study failed to provide valuable information on fluid responsiveness.

## Abbreviations

AUC = area under the receiver operating characteristic curve; CVP = central venous pressure; GEDV = global end-diastolic volume; PPV = pulse pressure variation; ROC = receiver operating characteristic; SV = stroke volume; SVV = stroke volume variation; SVV_FloTrac _= SVV assessed using FloTrac™/Vigileo™; SVV_PiCCO _= SVV assessed using PiCCO_plus_™.

## Competing interests

This study was supported in part by a research grant from Edwards Lifesciences (Irvine, CA, USA). In the past, the Institute of Anaesthesiology and Intensive Care Medicine, Triemli City Hospital have held research grants from Pulsion Medical Systems (Munich, Germany) and Edwards Lifesciences. CKH has received lecture fees from both Pulsion Medical Systems and Edwards Lifesciences in the past.

## Authors' contributions

CKH was responsible for study design and protocol, patient recruitment, measurements, data collection, statistical analysis and manuscript writing. AS was responsible for patient recruitment, measurements, data collection and manuscript writing. LW was responsible for patient recruitment, data collection and technical support. AZ was responsible for study design and protocol.
